# Spine Kinematics Behavior During the Handstand Posture: A Biplanar Radiographic Analysis

**DOI:** 10.3390/jfmk9040252

**Published:** 2024-12-03

**Authors:** Camille Eyssartier, Pierre Billard, Patricia Thoreux, Christophe Sauret

**Affiliations:** 1 Institut de Biomécanique Humaine Georges Charpak, Arts et Métiers Sciences and Technologies, 75013 Paris, France; 2 French Gymnastics Federation, 75010 Paris, France; 3 Institut de Biomécanique Humaine Georges Charpak, Université Sorbonne Paris Nord, 93430 Villetaneuse, France; 4 Centre d’Investigations en Médecine du Sport, Hôpital Hôtel Dieu, Assistance Publique-Hôpitaux de Paris, 75004 Paris, France; 5 Center for Research and Studies on Assistive Devices for People with Disabilities (CERAH), Institution Nationale des Invalides, 75007 Paris, France

**Keywords:** biomechanics, gymnastics, handstand, pelvis, spine, X-ray

## Abstract

**Background/Objectives:** The handstand is an exercise performed in many sports, either for its own sake or as part of physical training. Unlike the upright bipedal standing posture, little is known about the sagittal alignment and balance of the spine during a handstand, which may hinder coaching and reduce the benefits of this exercise if not performed correctly. The purpose of this study was to quantify the sagittal alignment and balance of the spine during a handstand using radiographic images to characterize the strategies employed by the spino-pelvic complex during this posture. **Methods:** Nineteen national-level artistic gymnasts participated in this study and underwent a low-dose biplanar (frontal and lateral) radiograph in both upright bipedal standing posture and during a handstand. Then, 3D reconstruction of the spine, based on biplanar radiographic images, enabled the determination of key pelvic (pelvic incidence, sacral slope, pelvic tilt) and spinal (lumbar lordosis, thoracic kyphosis, T9 sagittal offset) parameters in both postures. **Results:** The results showed that most gymnasts performed pelvic retroversion during the handstand, which was accompanied by an average decrease in lumbar lordosis, thoracic kyphosis, and T9 sagittal offset. Additionally, lumbar curvature was found to depend on pelvic orientation in upright bipedal standing posture, whereas it was associated with the thoracic spine during the handstand. **Conclusions:** This study provides new insights into how the spine kinematically adapts to an inverted body load. The results may help coaches and physiotherapists in teaching the handstand or using it to rehabilitate and strengthen the spine through the handstand posture.

## 1. Introduction

The sagittal alignment and balance of the spine in an upright bipedal standing posture has been extensively studied to understand how the body stays balanced when people exhibit various spino-pelvic parameters [1,2]. However, little attention has been given to the sagittal alignment of the spine during an inverted stance to explore the balance strategies employed in this non-physiological body orientation.

The handstand, a common posture in sports such as gymnastics and yoga, as well as in cross training, street workout, and in the physical conditioning for athletes across various disciplines, is widely recognized as an excellent core-strengthening exercise. In gymnastics, it is considered a foundational skill, essential for performing more advanced movements, serving as the starting point and conclusion of the integral components of many gymnastic movements [3].

In the scientific literature, several studies have investigated the strategies used to maintain a handstand, focusing on the motion of the center of mass and the center of pressure [4], muscles involvement [5], the role of vision [6], and the coordination of joints and body segments [7]. While the behaviors of the wrist, the shoulder, the hip, and the knee have been studied, no research has considered what happens between the hip and the shoulders, as if the trunk was a single rigid body [3,7]. It is all the more surprising given the trunk’s critical role in handstand performance, where its muscles would play a crucial contribution in maintaining balance and adjusting leg position.

In gymnastics, achieving a proper handstand requires a shoulder extension to align the arms, trunk, and legs [3]. Gaining a deeper understanding of the strategies for handstand balance, particularly the behavior of the spino-pelvic complex, could enhance both the learning and teaching of this posture while protecting spine health. Studying the spino-pelvic complex during a handstand also offers insights into how the spine adapts to an altered body orientation, a subject of potential interest to physiotherapists, as it may shed light on the functional anatomy of the spine.

Analyzing the sagittal alignment and balance of the spine during a handstand, as has been performed for an upright bipedal standing posture, requires a method for quantifying spino-pelvic parameters, particularly spinal curvature (i.e., the lumbar lordosis and the thoracic kyphosis), along with key pelvic position and orientation parameters (i.e., the pelvic incidence, the sacral slope, and the pelvic tilt) [1,2,8]. The T9 sagittal offset, currently used to describe spinal balance in an upright bipedal standing posture, could also be relevant [1]. To determine theses parameters in an upright bipedal standing posture, tri-dimensional (3D) reconstructions of the bones based on medical images such as EOS stereoradiographic system (EOS system, EOS imaging, Paris, France) have been of great interest [1,9]. The EOS system enables stereoradiographic images of the subject to be taken in frontal and lateral views simultaneously, with the subject being in a standing posture and with a low dose of irradiation. From 3D reconstructions of the pelvis and the thoraco-lumbar spine [10,11], parameters describing the sagittal balance of the spino-pelvic complex can be calculated with an accuracy ranging from 3° to 6° for the lumbar lordosis and thoracic kyphosis and from 1° to 3° for the pelvic position and orientation parameters (pelvic incidence, sacral slope, pelvic tilt) [11,12]. Although the use of the EOS system to study the spine during a handstand seems very challenging from an experimental perspective, as it would require that a subject maintain a handstand in the narrow EOS cabin while being very stable during the entire duration of the cliché (about 20 to 30 s in total), it would enable us to examine what is really happening at the spine level during the handstand.

Therefore, the aim of this study was to analyze the sagittal alignment and balance of the spine during the handstand using EOS stereoradiographic images, in comparison with the upright bipedal standing posture. It was hypothesized that the balance of the spine is achieved through a different sagittal alignment in upright bipedal standing and handstand postures.

## 2. Materials and Methods

### 2.1. Participants

After receiving ethical approval for the study (ID RCP: 2018-A01926-49; Comité de Protection des Personnes Sud Mediterranée V), 19 gymnasts (7 women and 12 men) were recruited between April 2019 and May 2022. All participants were involved in artistic gymnastics, trained more that fifteen hours per week and competed at the national level. The inclusion criteria required participants to be at least 12 years old and free from any pain or injury that could hinder their ability to train. The participants’ characteristics are detailed in Table 1.

### 2.2. Data Collection

**Figure 1:**
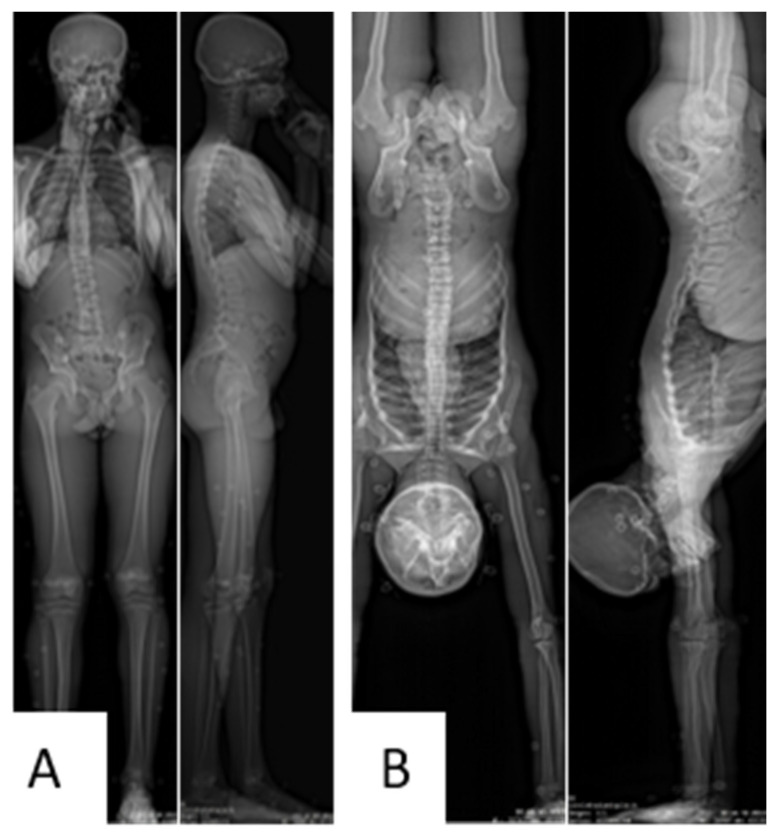
Example of a stereoradiographies (frontal and lateral views) of a gymnast in upright bipedal standing (**A**) and handstand (**B**) postures.

Each gymnast underwent low-dose biplanar (face and lateral) radiographs using the EOS system (EOS imaging, Paris, France) in both a standardized neutral standing posture (Figure 1A) and while performing a handstand (Figure 1B). To ensure stability during the handstand for the EOS scan (lasting 30 s in total: approximately 10 s for positioning within the acquisition volume, followed by about 20 s of scan itself), the gymnasts were asked to touch the cabin wall with one foot (it was asked that the ankle of this foot be placed in neutral position to allow for minimizing the deviation of the lower limb to only a few degrees). An instruction was also provided to the participants to minimize this contact force with the cabin wall.

### 2.3. Data Processing

**Figure 2:**
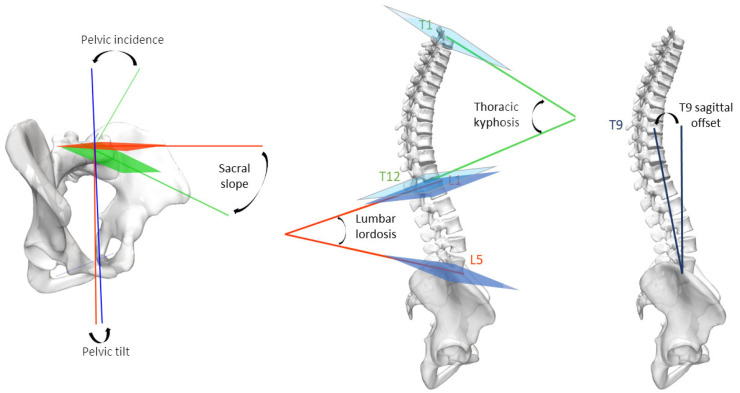
Illustration of the pelvic and spinal parameters considered in the analysis.

Based on EOS biplanar radiographs, 3D reconstructions of the pelvic bones and thoraco-lumbar vertebrae were performed by certified operators [9,10]. These 3D models enabled the calculation of key pelvic (pelvic incidence, sacral slope, pelvic tilt) and spinal (lumbar lordosis, thoracic kyphosis, T9 sagittal offset) parameters that characterize the sagittal spinal alignment and balance. For that purpose, a bone mesh was associated with a regionalization dataset for the bones of interest—i.e., pelvis and lumbar and thoracic vertebrae (more specifically, the pelvis and L5, L1, T12, T9, T4, T1 vertebrae)—allowing for a 3D coordinate system to be assigned to each bone and the parameters detailed in Table 2 and illustrated in Figure 2 to be calculated.

### 2.4. Analysis

After having checked the normal distribution of the data using Shapiro–Wilk tests, each spino-pelvic parameter was compared between the two postures using parametric paired samples *t*-test, with the significance level set at α = 5%.

Similar to previous studies on an upright bipedal standing posture [1], correlations between parameters for both postures were assessed using Pearson’s correlation coefficients (r). Coefficients ranging between ±0.70 and ±1 were considered as a strong correlation, and those ranging between ±0.40 and ±0.69 were considered as a moderate correlation.

Additionally, pelvic and spinal parameters were analyzed in order to identify different strategies used by the spino-pelvic complex during a handstand. For this analysis, gymnasts were categorized into two groups based on whether they displayed pelvic retroversion (decrease in sacral slope) or anteversion (increase in sacral slope) higher than the measurement uncertainty of the sacral slope (i.e., 4°) during the handstand. Participants exhibiting a change in the sacral slope within the range of measurement uncertainty were not included in any group. Non-parametric Mann–Whitney tests, with a significance threshold of 5%, were used to determine significant differences between the two groups.

The statistical analysis was performed using R software version 4.3.3 and the *p*-values (*p*) were reported. The Cohen effect size (d) was determined using G*Power software version 3.1.9.7.

## 3. Results

**Figure 3:**
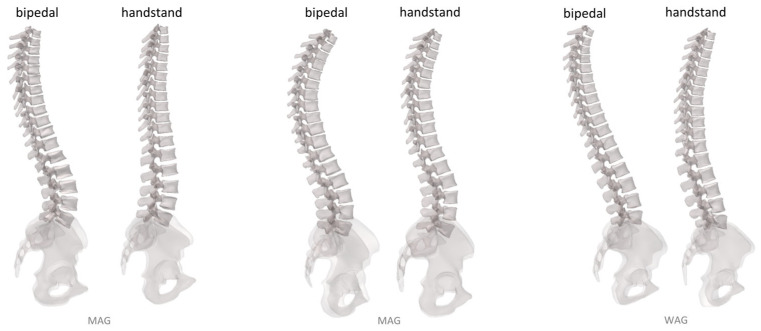
Illustration of the thoraco-lumbar spine of three subjects in bipedal and handstand postures.

The geometries of the pelvic bones and the thoracolumbar vertebrae were obtained in both postures for all 19 gymnasts. Figure 3 displays the spine of three gymnasts—selected for their differing spinal behavior—in the two postures of interest. For easier comparison, the spine during the handstand is flipped. This example highlights not only the changes in spinal curvature when performing a handstand but also the variations between individuals.

### 3.1. Sagittal Alignment of the Spine in the Bipedal and Handstand Positions

**Figure 4:**
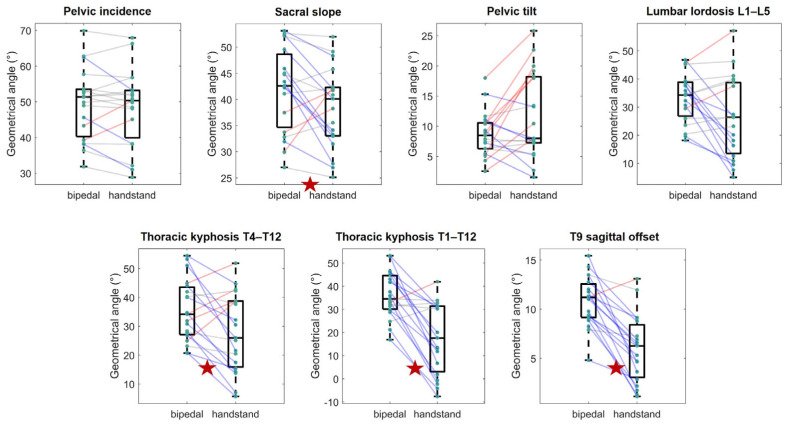
Whiskers box plots of spino-pelvic parameters in both upright bipedal standing and handstand positions. The bottom and top of the boxes represent the first and third quartiles, respectively; and the transverse line indicates the median. The ends of the whiskers show the maximum and minimum values. The green dots show individual values and the lines the individual variation (with a blue line meaning a decrease, a red line an increase, and a grey line a change considered not significant with regard to the measurement uncertainties). The red stars at the bottom of the graphs highlight significant differences between the two postures.

The results for all spino-pelvic parameters in both postures are shown in Figure 4. Significant differences between postures were observed for the sacral slope, lumbar lordosis, thoracic kyphosis (T4–T12 and T1–T12), and T9 sagittal offset. Specifically, compared to the upright bipedal standing posture, the handstand resulted in a decrease in the sacral slope (from 42° ± 8° to 38° ± 8° on average, *p* = 0.04, d = 0.49), along with an increase in the pelvic tilt (from 9° ± 4° to 12° ± 7° on average, *p* = 0.06, d = 0.46). Related to this pelvis retroversion, the results showed a decrease in the lumbar lordosis (from 33° ± 9° to 26° ± 14° on average, *p* = 0.02, d = 0.56) and both T4–T12 (from 35° ± 11° to 28° ± 14° on average, *p* = 0.01, d = 0.64) and T1–T12 thoracic kyphosis (from 36° ± 10° to 18° ± 15° on average, *p* < 0.001, d = 1.19), and a decrease in T9 sagittal offset (from 11° ± 3° to 6° ± 3° on average, *p* < 0.001, d = 1.44).

Pelvic incidence appeared to remain stable between the two postures (from 49° ± 10° to 48° ± 11° on average, *p* = 0.33, d = 0.10) although individual variations were observed, with changes ranging from 1° to 9°.

### 3.2. Correlation Between the Spino-Pelvic Parameters in the Bipedal and Handstand Positions

Table 3 presents the correlation matrix (Pearson r coefficients) for the spino-pelvic parameters in both postures. In the upright bipedal standing posture, strong correlations were observed between pelvic incidence and the sacral slope, as well as between the sacral slope and lumbar lordosis. Moderate correlations were found between pelvic incidence and both pelvic tilt and lumbar lordosis. During the handstand, only moderate correlations were observed, with the strongest correlations found between the sacral slope and both lumbar lordosis and thoracic kyphosis (both T1–T12 and T4–T12).

From the upright bipedal standing posture to the handstand, the correlations between the sacral slope and lumbar lordosis (from −0.73 to −0.67), and between pelvic tilt and pelvic incidence (from 0.59 to 0.56), remained relatively stable but were not highly correlated. In contrast, the correlation between lumbar lordosis and pelvic incidence decreased during the handstand (from r = 0.64 to r = 0.32), while the correlation between lumbar lordosis and thoracic kyphosis (both T4–T12 and T1–T12) increased noticeably (from r = 0.04 to r = 0.66). Additionally, the correlation between the sacral slope and thoracic kyphosis (both T4–T12 and T1–T12) also increased during the handstand (from r = 0.04 to r = 0.66 for both).

### 3.3. Strategies Used by the Spino-Pelvic Complex to Maintain a Handstand

**Figure 5:**
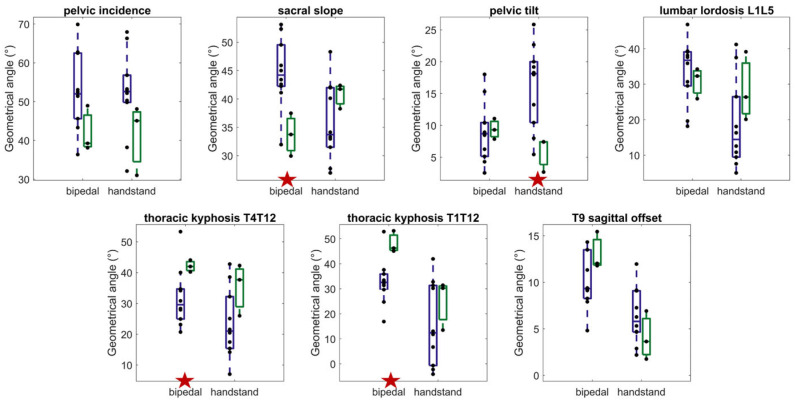
Pelvic and spinal parameters with gymnasts being assigned to two groups depending on whether they did a retroversion (Group 1 in blue—10 subjects) or an anteversion (Group 2 in green—3 subjects) of the pelvis during the handstand. The stars highlight significant differences between the two groups in either the bipedal or handstand postures). Dots represent individual values.

Although the average values indicate that gymnasts exhibited pelvic retroversion during the handstand, some gymnasts showed an increase in the sacral slope and a decrease in pelvic tilt, suggesting they adopted pelvic anteversion during the handstand. To differentiate between gymnasts performing a pelvic retroversion and those performing a pelvic anteversion, changes in the sacral slope were used with regard to the measurement accuracy (3°). The results showed that ten gymnasts did a pelvic retroversion (Group 1), three a pelvic anteversion (Group 2), and six fell into neither of these two groups because their pelvis remained in a relatively neutral position (with respect to the bipedal position, i.e., with a change lower than the measurement uncertainty). Figure 5 presents the pelvic and spinal parameters for the retroversion and anteversion groups.

The variation in the pelvic and spinal parameters between the upright bipedal standing and handstand postures were compared between the two groups. As expected from the definition of the groups, the variations of the sacral slope (*p* = 0.01) and pelvic tilt (*p* = 0.03) were significantly different between the two groups: Group 1 was characterized by a decrease in the sacral slope and an increase in pelvic tilt (pelvic retroversion) during the handstand, whereas Group 2 was characterized by an increase in the sacral slope and a decrease in pelvic tilt (pelvic anteversion). Group 2 also exhibited a significantly greater reduction in T9 sagittal offset (*p* = 0.05) during the handstand.

The pelvic and spinal parameters of these two groups were also compared in both upright bipedal standing and handstand postures. The results indicated significant differences in spino-pelvic sagittal alignment between the two groups, even from the upright bipedal standing posture. Gymnasts who adopted a pelvic anteversion during their handstand exhibited a significantly lower sacral slope (*p* = 0.03) but a significantly higher thoracic kyphosis T4–T12 (*p* = 0.05) and T1–T12 (*p* = 0.03) in an upright bipedal standing posture. Furthermore, their pelvic tilt was significantly lower during the handstand, likely due to the pelvic anteversion.

## 4. Discussion

In the current study, it was hypothesized that spinal balance is achieved through different sagittal alignments in upright bipedal standing and handstand postures. To test this hypothesis, spino-pelvic parameters of 19 gymnasts were calculated in the both postures using EOS low-dose biplanar radiographs and an associated 3D-reconstruction bone process. The orientation of the pelvis, the sagittal alignment of the spine, and the correlations between spino-pelvic parameters were then compared between the two postures.

The results showed that the sacral slope of the gymnasts significantly decreased during the handstand. Although the increase in pelvic tilt was not significant, the decrease in the sacral slope suggests that, on average, gymnasts performed pelvic retroversion during a handstand. However, the analysis of individuals revealed that while most of the gymnasts performed a pelvic retroversion during the handstand, three gymnasts performed a pelvic anteversion and six showed no noticeable change in pelvic orientation. Along with the trend of pelvic retroversion during the handstand, lumbar lordosis decreased, on average, compared to the upright bipedal standing posture.

Pelvic incidence appeared to remain stable, on average, between the two postures, although individual variations could be seen with amplitudes ranging from 1° to 9°. This parameter is described as physiological in the literature [1] and is generally assumed to be fixed for each individual. However, a radiographic study demonstrated that pelvic incidence changes with extreme anterior and posterior pelvic rotations compared to the neutral position [13]. These changes were found to exceed 5° in about 25% of the population for both maximal pelvic anteversion and retroversion [13]. The results of the present study also suggest that pelvic incidence may change with pelvis orientation, as 26% of gymnasts (i.e., five gymnasts) showed changes greater than 5° during the handstand, with no consistent pattern in the direction of these changes.

Regarding the thoracic spine, the handstand resulted in a significant decrease in T4–T12 and T1–T12 thoracic kyphosis, indicating a global reduction in spinal curvatures and leading to a significant decrease in T9 sagittal offset. In the upright bipedal standing posture, this last parameter indicates the position of the trunk’s center of mass. Thus, it was expected that the T9 sagittal offset would change during the handstand as the global body’s center of mass is higher, contributing to instability [3]. Furthermore, given the need for the alignment of the arms, trunk, and legs in the handstand, it can make sense that the T9 sagittal offset would decrease.

Studying correlations between the spino-pelvic parameters helps us understand how the spine achieves balance and what the key parameters for equilibrium are [1]. The levels of correlation found between spino-pelvic parameters in the upright bipedal standing posture were consistent with previously published correlations [1]. Comparing these correlation levels between upright bipedal standing and handstand postures highlighted the balance mechanisms in a handstand. The results showed that in the upright bipedal standing posture, lumbar curvature depended on pelvic orientation. However, in the handstand, lumbar curvature depended more on the thoracic spine, probably because the “base of the trunk” was the thoracic spine rather than the pelvis in this inverted stance. In line with this observation, it should be noted that the angle between the trunk and the upper limbs was not measured, although the behavior of the thoracic spine may be affected by it and, subsequently, that of the lumbar spine.

The second hypothesis explored in the present study was that different strategies can be employed by the spino-pelvic complex during a handstand. Based on spino-pelvic parameters, two strategies were identified, depending on whether the gymnasts performed pelvic anteversion or retroversion during the handstand. To test this second hypothesis, sagittal alignment and balance of the spine were compared between gymnasts using one of these two strategies. Most gymnasts performed a pelvic retroversion during the handstand, likely in line with instruction typically given when learning the handstand. However, 16% of gymnasts (3 gymnasts) performed a pelvic anteversion and 32% (6 gymnasts) maintained a pelvis orientation close to its neutral position. There was no correlation with gender, gymnastics discipline, or head position, but it appeared that some spino-pelvic parameters were already different in the bipedal position between the two groups (pelvic retroversion vs anteversion), which may explain the different strategies of the spino-pelvic complex during the handstand. Indeed, gymnasts who performed a pelvic anteversion during the handstand had a more vertical pelvis in an upright bipedal standing posture (significantly lower sacral slope compared to the group exhibiting a pelvic retroversion during the handstand). A more vertical pelvis leaves less scope for retroversion, which may explain why they used pelvic anteversion. Also, gymnasts performing a pelvic anteversion during the handstand exhibited significantly higher thoracic kyphosis in the upright bipedal standing posture, which may explain the greater reduction in T9 sagittal offset observed in this group between the upright bipedal standing and handstand postures.

From a muscular perspective, the handstand requires isometric contractions of abdominal, gluteal, and back muscles to achieve an optimal body alignment [3,5]. Yet, it has been shown that erector spinae activity was more pronounced with pelvic anteversion, while transversus abdominis activity was greater with pelvic retroversion [14,15]. Although these results apply to the upright bipedal standing posture, it would be interesting to investigate whether a pelvic retroversion strategy during the handstand leads to greater transversus abdominis activation, as this core muscle plays a key role in body stabilization. This hypothesis aligns with gymnastics practice, where pelvic retroversion is recommended during the handstand training to improve trunk stability and, to a lesser extent, protect the lumbar spine. Therefore, it would be valuable to estimate intervertebral stresses during the handstand to assess whether pelvic retroversion actually reduces the spinal stress.

### Limitations

Although this study is the first to assess the sagittal alignment and balance of the spine during a handstand, several limitations should be noted. First, because the stereoradiographic imaging requires the gymnast to remain completely still, gymnasts were asked to lightly press the tips of their toes—on one leg—against a wall of the EOS cabin to ensure immobility. This contact would favor a decrease in lumbar lordosis. However, precautions were taken to ensure this adjustment did not alter the handstand or significantly impact spinal alignment (due to the low global alignment angle from the vertical and the instructions provided to the participants to minimize this contact force). However, it was the only practical way to obtain radiographic images of the spine in this posture using the available technology. Second, when distinguishing between gymnasts performing pelvic retroversion and anteversion during the handstand, only three gymnasts fell into the anteversion group (Group 2). Including more gymnasts in this group, by including more gymnasts beforehand, could have strengthened the conclusions about this group’s spinal behavior compared to the retroversion group (Group 1), providing more insights into why some gymnasts employed a pelvic anteversion. Third, a larger sample size would also allow for an assessment of whether gymnasts develop different strategies for performing the handstand based on their type of back, according to the Roussouly classification [16,17], for instance. Finally, the handstand is not always a static posture, and it would be interesting to investigate how the spine adjusts to inertial loading, and not only during static positions. Unfortunately, this question could not be addressed using the methodology employed in this study, namely stereoradiographic imaging. The use of other technologies, such as motion capture and inertial measurement units, may be helpful for this purpose. However, such tools would first need to demonstrate their ability to provide enough accuracy, in particular considering that such technologies are subjected to soft-tissue artifact.

## 5. Conclusions

The aim of this study was to analyze the sagittal alignment and balance of the spine during the handstand, compared to an upright bipedal standing posture, using EOS low-dose biplanar radiographs. Based on these original images, this study provides valuable insights for physiotherapists as well as medical and technical staff. The results demonstrate that the handstand leads to an overall reduction in spinal curvatures. These changes in spinal curvatures are expected to be due to deep core muscles, in particular to erector spinae and transversus abdominis muscles. Hence, strengthening these muscles through core muscle exercises can favor good execution of the handstand. From another perspective, with proper instructions, the handstand can also be considered as a good exercise to strengthen the spine muscles. However, it must be noted that this study focused on a handstand performed by gymnasts, and the results may be different if other populations are enrolled. Evaluating the different ways of performing a handstand from the methodology used in this study is a perspective. Additionally, the findings of this study also highlight the importance of considering pelvic tilting during the handstand, particularly noting that athletes with a more vertical sacral slope and a greater thoracic kyphosis may tend to adopt pelvic anteversion to maintain the spinal balance during the handstand. With further research on this aspect, it may be possible to tailor handstand training according to the athletes’ spinal profiles.

Finally, this study contributes both to identifying the underlying mechanisms of the spino-pelvic complex during a handstand and to improving the understanding of how the spine adapts to altered position and loading. This dual objective should aid gymnastics coaches in teaching the handstand and assist physiotherapists in rehabilitating the spine function.

## Figures and Tables

**Table 1:** Participants’ ages and anthropometrics (mean ± SD, [range]).

	Female	Male
Age (years old)	17 ± 2 [13–19]	15 ± 3 [12–21]
Height (cm)	154 ± 6 [148–161]	156 ± 10 [140–173]
Mass (kg)	51 ± 6 [45–60]	52 ± 12 [38–80]

**Table 2:** Description of the spino-pelvic parameters quantified in this study.

Parameters	Description
Pelvic incidence	Angle between the line perpendicular to the sacral endplate plane and the line joining the middle of the sacral endplate and the midpoint of the bicoxofemoral axis.
Sacral slope	Angle between a horizontal plane and the plane of the sacral endplate.
Pelvic tilt	Angle between the vertical axis and the line joining the middle of the sacral endplate and the midpoint of the bicoxofemoral axis.
Lumbar lordosis L1–L5	Angle between the plane of the cranial endplate of the first lumbar vertebra (L1) and the plane of the caudal endplate of the fifth lumbar vertebra (L5).
Thoracic kyphosis T4–T12	Angle between the plane of the cranial endplate of the fourth thoracic vertebra (T4) and the plane of the caudal endplate of the twelfth thoracic vertebra (T12).
Thoracic kyphosis T1–T12	Angle between the plane of the cranial endplate of the first thoracic vertebra (T1) and the plane of the caudal endplate of the twelfth thoracic vertebra (T12).
T9 sagittal offset	Angle between the vertical plumb line and the line joining the center of the vertebral body of the ninth thoracic vertebra (T9) and the midpoint of the bicoxofemoral axis.

**Table 3:** Matrix of correlation (Pearson’s r coefficients) between spino-pelvic parameters * in upright bipedal standing (BP) and handstand (HS) postures. The upper part (white background) of the matrix is related to the upright bipedal standing posture and the lower part (light gray background) to the handstand. Coefficients ranging between ±0.70 and ±1 indicate a strong correlation (highlighted in bold); those ranging between ±0.40 and ±0.69 indicate a moderate correlation (highlighted in bold and italic).

Parameters	PI	SS	PT	LL	TK4–12	TK1–12	T9 Offset
PI		**−0.89**	***0.59***	***0.64***	−0.03	0.16	−0.23
SS	***−0.54***		−0.22	**−0.73**	0.04	−0.15	0.38
PT	***0.56***	0.29		0.19	−0.16	0.00	0.32
LL	0.32	***−0.67***	−0.25		−0.14	−0.03	0.02
TK4–12	−0.11	***0.66***	0.28	***−0.49***		**0.78**	−0.36
TK1–12	−0.38	***0.65***	0.00	***−0.45***	**0.85**		***−0.50***
T9 offset	0.06	0.11	0.35	0.17	−0.36	***−0.44***	

* PI: pelvic incidence; SS: sacral slope; PT: pelvic tilt; LL: lumbar lordosis L1–L5; TK4–12: thoracic kyphosis T4–T12; TK1–12: thoracic kyphosis T1–T12; T9 offset: T9 sagittal offset.

## Data Availability

The original contributions presented in this study are included in the article. Further inquiries can be directed to the corresponding author(s).
